# Metabolic Characteristics of PGPR-Induced Growth Promotion in Alfalfa (*Medicago sativa* L.)

**DOI:** 10.3390/metabo15100652

**Published:** 2025-09-30

**Authors:** Rina Dao, Ying Zhang, Qiang Li, Shengyan Lei

**Affiliations:** Key Laboratory of Alpine Grassland Ecology in the Three Rivers Source Region, Qinghai Provincial Key Laboratory of Adaptive Management on Alpine Grassland, College of Agriculture and Animal Husbandry, Qinghai University, Xining 810016, China; zhangying2019@126.com (Y.Z.);

**Keywords:** PGPR, *Medicago sativa*, metabolomics, KEGG enrichment, isoflavonoid biosynthesis

## Abstract

**Background/Objectives**: Plant growth-promoting rhizobacteria (PGPR) have demonstrated potential for enhancing plant growth; existing research inadequately characterizes the metabolic underpinnings of PGPR-induced plant phenotypes. **Methods**: A deeper investigation into the impact of PGPR on plant metabolic pathways is crucial for a comprehensive understanding of their growth-promoting mechanisms and for the development of more effective biofertilizers and plant protection strategies. **Results**: To clarify the core metabolic pathways targeted by PGPR strains, we selected alfalfa as the research object, employed two *Pseudomonas* combinations, and utilized a broad-targeted metabolomics approach to investigate the metabolic characteristics of alfalfa roots. Through the analysis of primary and secondary metabolites, a total of 2694 metabolites were identified, among which lipids were the main nutrients during the growth of alfalfa. The L-citrulline and L-arginine contents were significantly upregulated, thereby affecting nitrogen metabolism and ultimately promoting plant growth. In addition, different branches of the isoflavonoid biosynthesis pathway showed differential regulation, indicating their close relationship with plant growth promotion. **Conclusions**: This study provides a new perspective for a deeper understanding of the molecular mechanisms by which PGPR promotes plant growth and lays a theoretical foundation for the future development of PGPR-based agricultural biological agents.

## 1. Introduction

The rhizosphere, a narrow zone surrounding and influenced by plant roots, harbors a diverse microbial community that is crucial for plant growth, nutrient acquisition, and overall health [1,2]. Plant growth-promoting rhizobacteria (PGPR) are rhizosphere bacteria that can enhance plant growth and health. They can directly promote plant development by modulating plant hormone levels, such as abscisic acid, auxins, gibberellins, ethylene, and cytokinins, or by increasing the availability of essential mineral nutrients such as Fe, N, and P [2]. Furthermore, PGPR indirectly promotes plant growth by controlling detrimental microorganisms or pathogens, thereby preventing or mitigating the harmful effects of phytopathogens and environmental stressors on plants [3,4,5]. The reintroduction of 2–5% of rhizosphere bacteria through plant inoculation into soil with a competitive microbial community has positively impacted plant productivity [6]. Consequently, PGPR have been extensively studied for their potential applications in bioaugmentation, biocontrol, and biostimulation, offering nutrients, alleviating biotic and abiotic stresses, and secreting phytohormones and siderophores [7,8,9]. They represent a powerful tool for achieving sustainable agriculture by reducing or eliminating the use of pesticides and/or fertilizers without compromising yields [10].

Alfalfa, a perennial herbaceous legume, is renowned for its high yield of dry and fresh forage and its rich nutritional composition [10]. The leaves are tender and succulent, offering excellent palatability and digestibility, making it a premium forage for ruminants such as cattle and sheep, and earning it the reputation as the “king of forages” [11,12]. The root system is closely linked to soil physicochemical properties, and its extensive root network contributes to improved soil nutrient status. Furthermore, the exhibits ecological adaptability, including drought tolerance, cold hardiness, and salt–alkali resistance, plays a crucial role in soil and water conservation and sand fixation [13,14,15]. Alfalfa is preferentially selected for ecological projects such as saline–alkali land improvement, returning farmland to grassland, and wind and sand control [16,17,18]. Currently, research on alfalfa primarily focuses on cultivation and breeding [19,20], genetic diversity [19,21] and stress resistance [22]. There is a paucity of research on inoculating PGPR strains to induce alfalfa growth and exploring their promoting effects and metabolic mechanisms. In-depth research on the regulatory effects of PGPR strains on the overall metabolome of alfalfa, particularly on key metabolic pathways that promote growth, and elucidating how PGPR regulates specific metabolic pathways to promote plant phenotypes or enhance the synthesis of high-value plant compounds will provide important theoretical [23] support for a sustainable cultivation technology system for high-yield, high-quality, and efficient alfalfa production.

Through broad-spectrum targeted metabolomics analysis, we compared the metabolic alterations in alfalfa roots induced by distinct *Pseudomonas* strains and performed differential analysis against a control group (without inoculation). This approach aimed to identify the core metabolic pathways targeted by PGPR. The ultimate goal is to provide a theoretical basis for the directed regulation of plant metabolism using PGPR strains, thereby promoting the biosynthesis of target natural products.

## 2. Materials and Methods

### 2.1. Test Materials

Alfalfa seeds were stored in the experimental building of the College of Agriculture and Animal Husbandry, Qinghai University, China (Courtesy of Professor Wenhui Liu, Institute of Animal Husbandry and Veterinary Science, Qinghai University). The N-*Pseudomonas* sp. (JQ435712.1) and OP-*Pseudomonas chlororaphis* (MT102292.1) strains were obtained from the isolation and screening of rhizosphere soil of dominant plants in alpine wetlands (101°57’~102°06′ E, 34°12′~34°32′ N) in the early stage of the laboratory (Tables S1–S3), and there was no antagonistic reaction between the two strains.

### 2.2. Preparation of Bacterial Suspension

The test strains were inoculated into 50 mL of sterile LB liquid medium [24] and cultured at 28 °C and 125 rpm/min for 48 h. The cultured bacterial cells were collected by centrifugation (10,000 rpm/min, 4 °C, 5 min), the supernatant was discarded, and the bacterial cells were resuspended in sterile water to prepare the bacterial suspension. The composite bacterial suspensions were mixed in equal proportions and concentrations (the concentration of bacterial suspension was calculated by using the absorbance of the spectrophotometer at 600 nm; the concentration of bacterial suspension of each strain was adjusted to 1 × 10^8^ cfu/mL with sterile water to ensure that they were at equal concentration).

### 2.3. Preparation of Plant Pot Experiments

Alfalfa seeds were disinfected with 70% ethanol for 1 min and rinsed with sterile water 3–5 times. Pot experiments were conducted in the College of Agriculture and Animal Husbandry, Qinghai University, from September to October 2024, using an artificial climate incubator with a light/dark cycle of 12 h/12 h, a temperature of 25–28 °C, and a relative humidity of 65%. The potting soil was prepared by mixing field soil and nutrient soil in a ratio of 2:1, with 400 g per pot, sterilized before planting. Nutrient composition of potted soil: total nitrogen 3.90 g/kg; total phosphorus 1.75 g/kg; total potassium 25.43 g/kg; alkaline nitrogen 274.33 mg/kg; available phosphorus 17.87 mg/kg; available potassium 216 mg/kg; organic matter 64.67 g/kg; pH 7.66. Every 5 d, 20 mL of bacterial suspension was applied to the roots of the plants, and the control group was treated with an equal amount of sterile water [13]. MN is the treatment group induced by N-*Pseudomonas* sp. strain; MP is the treatment group induced by OP-*Pseudomonas chlororaphis* strain; MP–N is the treatment group induced by two strains mixed; CK is the treatment group without strain induction and only irrigated with sterile water. Each treatment was repeated 3 times.

**Figure 1:**
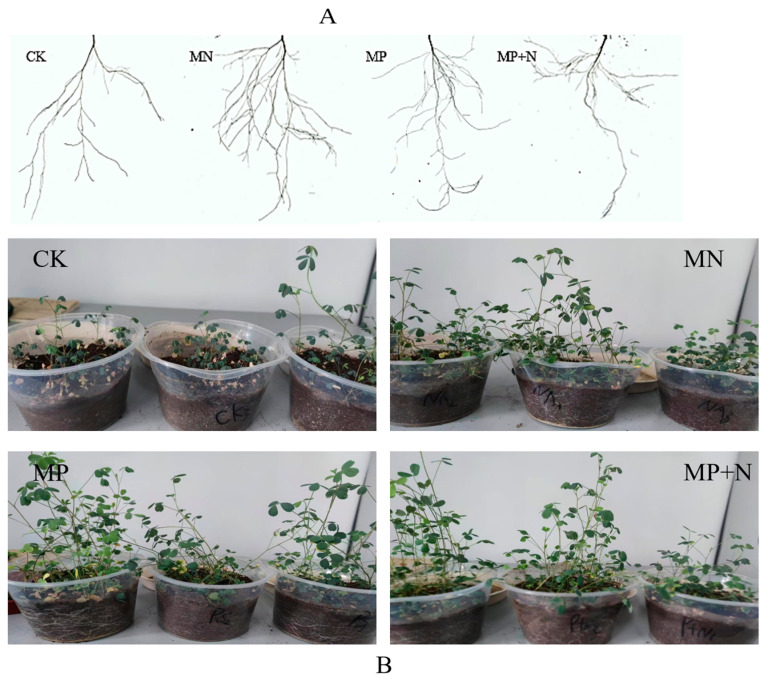
Preliminary experimental images: (**A**). root system morphology of alfalfa under different treatments; (**B**). potting experiment of alfalfa under different treatments.

The plants were harvested 40 d after sowing (before blooming) (Figure 1A,B), and the plant height, aboveground, and underground fresh weight were measured (3 plants were randomly selected from each pot, and the average value was taken) (File S1). The plant roots were washed with distilled water and scanned using a scanner (Deskscan System-Root Law Program, USA) to record the total root length, root volume, root surface area, and average root diameter. The plant roots were collected (each process handles eleven iterations/roots), placed in cryopreservation tubes, and quickly frozen in liquid nitrogen for subsequent sample extraction.

### 2.4. Metabolomics Assay Methods

Following lyophilization, 50 mg of the sample was weighed and mixed with 1000 μL of extraction solvent (methanol: acetonitrile: water, 1:2:1, *v*/*v*/*v*). The mixture was vortexed for 30 s. After adding steel beads, the sample was homogenized using a 45 Hz grinder for 10 min, followed by 10 min of sonication in an ice–water bath. The sample was then incubated at −20 °C for one hour. Subsequently, the sample was centrifuged at 4 °C, 12,000 rpm for 15 min. A total of 300 μL of the supernatant was carefully collected and filtered through a 0.22 μm organic filter membrane into a 2 mL autosampler vial. A 10 μL aliquot of each sample was pooled to create a quality control (QC) sample for instrumental analysis. The analytical platform comprised a Waters Acquity I-Class PLUS UPLC system coupled to an AB Sciex Qtrap 6500+ mass spectrometer, configured for high-sensitivity detection. Sample analysis was performed according to the established parameters.

### 2.5. Data Analysis

Metabolite annotation was performed using the KEGG database [25] (http://www.genome.jp/kegg/) (3 March 2025). PCA and three-dimensional pie chart illustrating the composition of metabolite classes were generated using R version 3.6.1. Enrichment analysis was performed using the cluster Profiler and enrichplot (versions 4.4.4 and 1.2.0, respectively) packages. K-means clustering was performed using the k-means function from the R base package, along with NbClust (versions 3.6.1 and 3.0) to determine the optimal number of clusters. Data were scaled using UV scaling. MN is the treatment group induced by N-*Pseudomonas* sp. strain; MP is the treatment group induced by OP-*Pseudomonas chlororaphis* strain; MP–N is the treatment group induced by two strains mixed; CK is the treatment group without strain induction and only irrigated with sterile water.

## 3. Results

### 3.1. Orthogonal Partial Least Squares Discriminant Analysis

**Figure 2:**
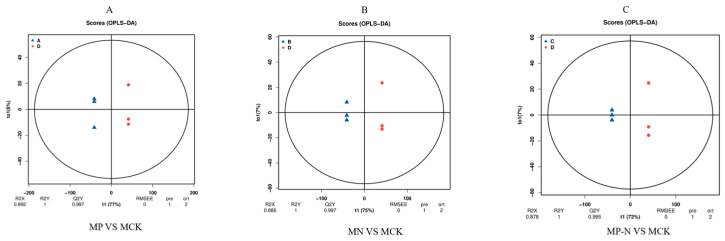
(**A**) MP vs MCK; (**B**) MN vs MCK; (**C**) MP-N vs MCK; OPLS-DA score plot. The *x*-axis (t1) represents the predictive component (between-group variance), and the *y*-axis (to1) represents the orthogonal component (within-group variance). The *y*-axis percentage indicates this component’s proportion in the total variance. The parameters of the model are noted below the figure, including R2X, R2Y, Q2Y, RMSEE (root mean square error), pre (number of predictive components), and ort (number of orthogonal components). The *x*-axis in the figure represents the correlation between the permutation groups and the original model groups, and the *y*-axis represents the values of R2Y or Q2Y (where the R2Y and Q2Y at *x*-axis value 1 are the values of the original model). The blue and red dots represent the R2Y and Q2Y of the permuted models, respectively, and the two dashed lines are the regression lines fitted for R2Y and Q2Y.

The OPLS-DA analysis results demonstrated a complete separation of the four sample groups in the OPLS-DA score and model plots (Figure 2A–C). The scatter plots corresponding to each group exhibited intra-group clustering, indicating high reproducibility and similarity within the samples, while also demonstrating good inter-group discrimination.

### 3.2. Description of Material Detection Results

**Figure 3:**
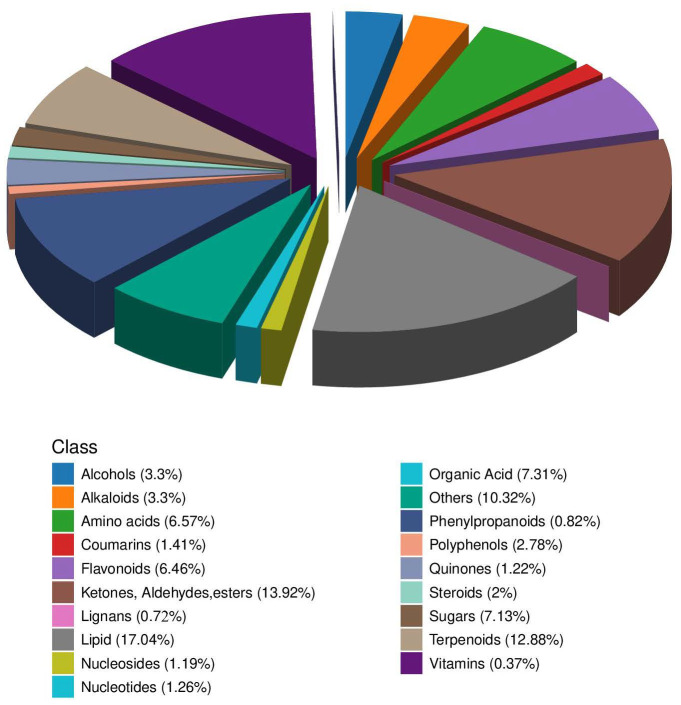
Three-dimensional pie chart illustrating the composition of metabolite classes. The drawing was carried out using the R language software (3.6.1); each color represents a different metabolic category, and the area of the color block indicates the proportion of that category.

To elucidate the patterns of metabolite alterations across diverse treatments, we employed a broad-target metabolomics approach using an LC-QTRAP platform to identify both primary and secondary metabolites within the samples (Figure 3). This analysis resulted in the detection of 2694 metabolites, including 19 primary metabolite classes and 40 secondary metabolite classes. Notably, lipids constituted 17.04% of the detected metabolites, with fatty acids, fatty acyls, isoprene lipids, and steroids being the predominant secondary metabolites identified. Ketones, aldehydes, and esters accounted for 13.92%, with polyketides and related compounds being the major secondary metabolites. Terpenoids represented 12.88%, primarily comprising triterpenoids, sesquiterpenoids, monoterpenes, and diterpenes.

### 3.3. Principal Component Analysis (PCA)

**Figure 4:**
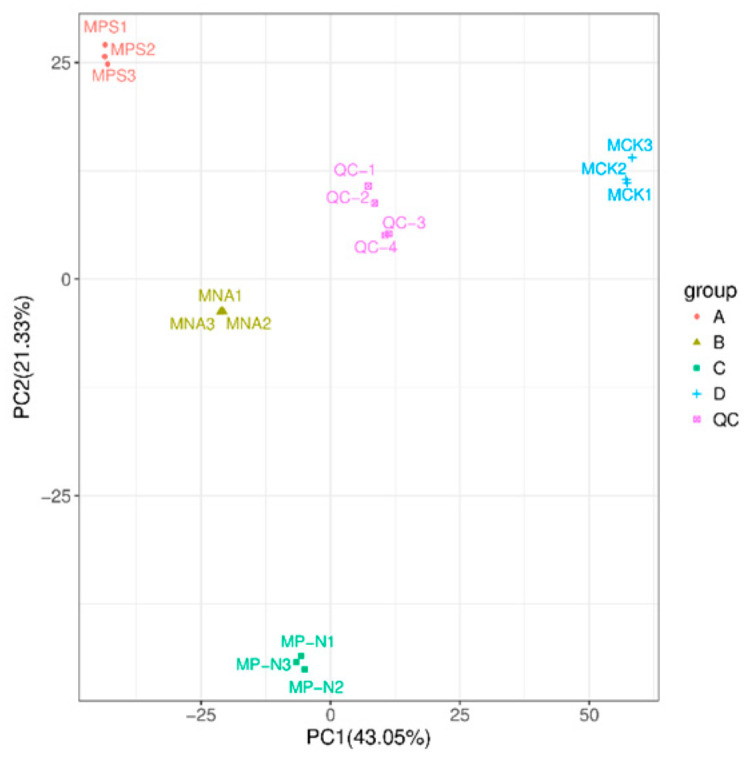
PCA analysis of all samples. The *x*-axis represents the first principal component, and the *y*-axis represents the second principal component. Each point in the figure represents a sample, with samples from the same group represented by the same color. Samples from different groups are labeled with different colors.

Metabolite PCA analysis (Figure 4) revealed a clear separation between the MP, MN, MP–N treatment groups and the control group (CK), indicating significant differences in metabolites among the four groups, with good clustering and high discrimination of the samples (Table S1). The first principal component (PC1) explained 43.05% of the variance in the original dataset, and the separation of MP–N, MN from MP, and CK treatments was observed on the first principal component. On the second principal component, CK was separated from the other three treatment groups, and this principal component explained 17.2% of the variance in the original dataset.

### 3.4. Screening and Identification of Differential Metabolites

**Figure 5:**
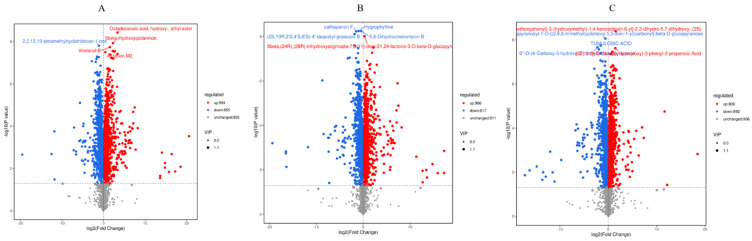
(**A**) MP vs MCK; (**B**) MN vs MCK; (**C**) MP-N vs MCK; Volcano plot of differential metabolites. Blue dots represent downregulated, differentially expressed metabolites, red dots represent upregulated, differentially expressed metabolites, and gray dots represent metabolites detected but with no significant difference. In addition, the top 5 qualitatively identified metabolites, ranked by *p*-value, are labeled in the figure.

Employing a dual-filter approach, we identified differentially abundant metabolites by integrating variable importance in projection (VIP) values > 1 with a fold change (FC) threshold of ≥2 or ≤0.5 (*t*-test). Figure 5 illustrates the distribution of significantly altered metabolite classes across the experimental groups. Metabolomic analysis revealed significant alterations in metabolite profiles across the treatment groups. In the MP vs. CK comparison, 1839 metabolites exhibited significant changes, indicating extensive metabolic divergence between the MP and CK groups. Specifically, 984 metabolites were upregulated, while 855 were downregulated. The slightly higher number of upregulated metabolites suggests the potential activation of certain metabolic pathways in the MP group, leading to increased levels of related metabolites. Key metabolites, as determined by *p*-value ranking, included Octadecanoic acid, hydroxy-, ethyl ester and 6β-Hydroxyipolamilde. These metabolites may play crucial roles in this comparison, and their significant changes in abundance likely correlate with physiological or pathological differences between the MP and CK groups. Further investigation is warranted to elucidate the biological functions of these metabolites and their roles in the relevant biological processes.

The MN vs. CK comparison identified significant changes in 1783 metabolites, a slightly smaller number than observed in the MP vs. CK group, yet still indicative of notable metabolic differences between the MN and CK groups. The analysis revealed 966 upregulated and 817 downregulated metabolites, with the numbers being relatively close, reflecting the complexity of metabolic changes in the MN group compared to the CK group. The top-ranked metabolites, based on *p*-value, included cathayanon F, Hygrophylline, and 5,6-Dihydrocineromycin B. These metabolites may serve as important markers of metabolic differences between the MN and CK groups, and their in-depth study could help reveal the fundamental metabolic distinctions between these groups.

In the MP–N vs. CK group, 1698 metabolites showed significant changes. Although the number was slightly reduced compared to the previous two groups, metabolic differences were still observed between the MP–N and CK groups. The analysis revealed 892 upregulated and 806 downregulated metabolites, with the number of upregulated metabolites slightly exceeding the number of downregulated metabolites, similar to the previous two groups.

In conclusion, all three groups exhibited a trend of upregulated metabolites exceeding downregulated ones (MP 53.5%, MN 54.2%, MP–N 52.5%), suggesting that the treatments may universally activate specific metabolic pathways (e.g., energy metabolism or synthetic pathways). The MN group displayed the highest upregulation ratio, implying the most potent metabolic activation effect; the MP–N group showed the lowest ratio, potentially due to antagonism or compensatory mechanisms between treatments.

### 3.5. Differential Metabolite Analysis

#### 3.5.1. K-Means Analysis of Differential Metabolites

**Figure 6:**
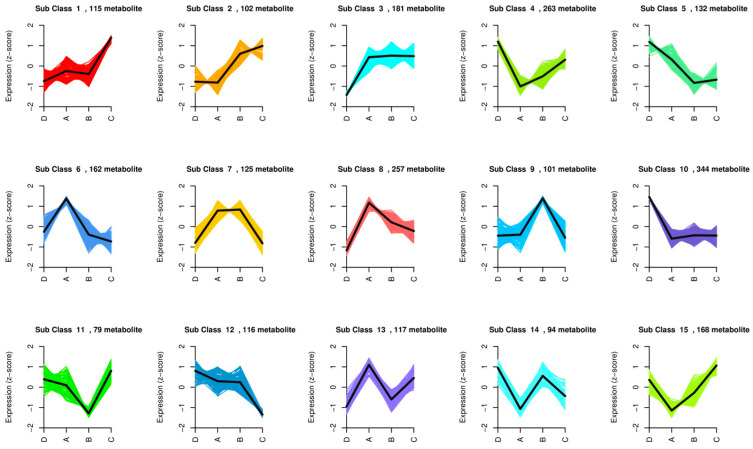
K-means clustering trend chart of differential metabolite set. The *x*-axis represents the grouping, and the *y*-axis represents the quantitative value of metabolites. Different colored lines represent the average change trend of the content of each k-means clustered metabolite between groups; Group A indicates MP treatment; Group B indicates MN treatment; Group C indicates MP–N treatment; Group D indicates CK treatment.

K-means clustering, originating from a vector quantization method in signal processing, partitions *n* data points (e.g., *n* metabolites) into *k* clusters, ensuring each point belongs to the cluster with the nearest mean (the cluster center), which serves as the clustering criterion. This method segregates metabolites with similar trends in abundance changes into distinct clusters, facilitating the investigation of functional similarities or direct/indirect regulatory relationships among metabolites within each cluster. A K-means clustering trend plot of differential metabolites is presented (Figure 6). K-means clustering of differential metabolites revealed 15 subgroups with similar variation trends. Subgroups 3, 4, 8, and 10 showed the highest number of differential metabolites (181–344). In subgroups 1 and 2, metabolite content was elevated in the MP–N treatment, with key metabolites including nucleotides, sugars, and polyphenols. Subgroups 4, 5, 10, and 12 exhibited higher metabolite content in the CK treatment, encompassing organic acids, flavonoids, amino acids, alcohols, and polyphenols. In subgroups 6, 8, and 13, the MP treatment showed significantly higher metabolite content, including amino acids, organic acids, lipids, terpenes, and polyphenols. Distinct treatments induce differential metabolite accumulation by modulating specific metabolic pathways, such as nucleotide synthesis, flavonoid biosynthesis, and lipid metabolism, reflecting the unique physiological effects of each treatment.

#### 3.5.2. KEGG Functional Annotation and Enrichment Analysis of Differential Metabolites

**Figure 7:**
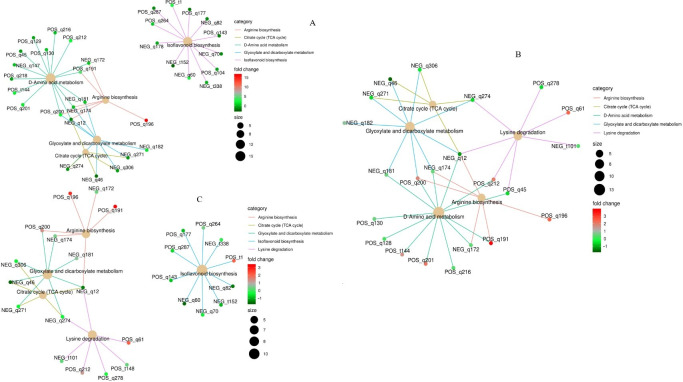
KEGG enrichment network of differential metabolites. In the figure, the light yellow nodes represent pathways, and the small nodes connected to them are the specific metabolites annotated to these pathways. The size of the nodes indicates the number of differential metabolites annotated to the pathway, and the color depth represents the log2 value of the fold change. (**A**): MP vs. CK; (**B**): MN vs. CK; (**C**): MP–N vs. CK.

Differential metabolites interact within biological systems, forming distinct pathways. KEGG database annotation of these differential metabolites was performed, and pathway entries with the highest number of annotated differential metabolites were selected to construct a summary network diagram (Figure 7). In MP vs. CK (Figure 7A), five target pathways with significant impact from differential metabolites were identified: Arginine biosynthesis, Citrate cycle (TCA cycle), D-Amino acid metabolism, Glyoxylate and dicarboxylate metabolism, and Isoflavonoid biosynthesis. L-Citrulline exhibited a fold change of 16.38 within the Arginine biosynthesis pathway, indicating upregulation. In MN vs. CK (Figure 7B), five target pathways with significant impact from differential metabolites were identified: Arginine biosynthesis, Citrate cycle (TCA cycle), D-Amino acid metabolism, Glyoxylate and dicarboxylate metabolism, and Lysine degradation. Upregulation was observed for L-Lysine (2.04-fold), L-Histidine (2.16-fold), L-Glutamine (1.85-fold), L-Citrulline (2.43-fold), L-Arginine (3.85-fold), Allysine (6-Oxo Dl-Norleucine) (2.45-fold), and 1-Pyrroline-4-hydroxy-2-carboxylic acid (1.69-fold). In MP-N vs. CK (Figure 7C), five target pathways with significant impact from differential metabolites were identified: Arginine biosynthesis, Citrate cycle (TCA cycle), D-Amino acid metabolism, Glyoxylate and dicarboxylate metabolism, and Lysine degradation. Upregulation was observed for Medicarpin 3-O-glucoside-6′-malonate (2.10-fold), L-Citrulline (2.77-fold), L-Arginine (3.31-fold), and Allysine (6-Oxo Dl-Norleucine) (2.15-fold).

### 3.6. Functional Analysis of Differential Metabolites in Each Treatment Group

**Figure 8:**
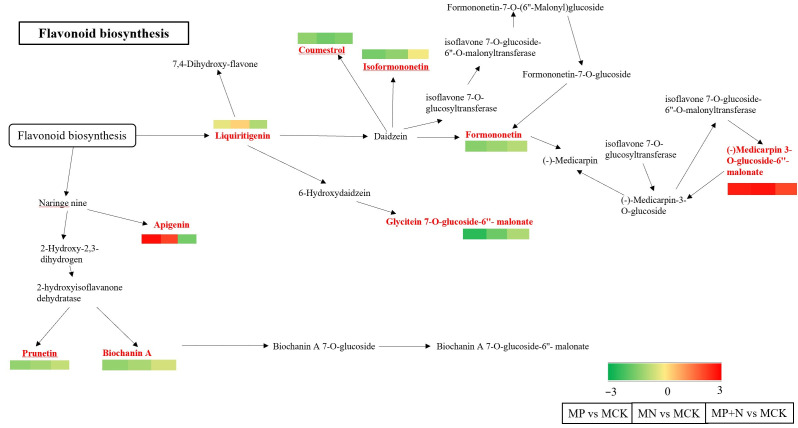
KEGG annotation results for differential metabolites. Red indicates significantly upregulated metabolite content.

Based on the previous analysis, the shared differential metabolic pathway among all groups was identified as Flavonoid biosynthesis within the Biosynthesis of other secondary metabolites pathway [26] (Figure 8). Several differential metabolites were commonly observed in this pathway across all groups, including significantly increased levels of Formononetin, Isoformononetin, Biochanin A, Apigenin, 6″-O-Malonylglycitin, and Medicarpin 3-O-glucoside-6′-malonate. The primary compounds involved in the upregulation of these metabolites were Formononetin, Malonylglycitin, Biochanin A, Apigenin 4′,5,7-Trihydroxyflavone, Isoformononetin 4′-Hydroxy-7-methoxyisoflavone, and Medicarpin 3-O-glucoside-6′-malonate.

Formononetin, a key upregulated metabolite, may regulate the pathway, triggering a cascade effect. Malonylglycitin may be a crucial intermediate in Isoflavonoid biosynthesis, influencing related metabolite synthesis. Biochanin A may modulate metabolite synthesis via cell signaling or gene expression. Apigenin may be involved in antioxidant defense and pathway regulation. Isoformononetin derivatives may regulate Isoflavonoid biosynthesis. Medicarpin derivatives may regulate metabolic flux and metabolite levels.

## 4. Discussion

### 4.1. Key Nutritional Components in the Growth of Alfalfa

In this study, a total of 2694 metabolites were identified during the growth of PGPR-induced/uninduced alfalfa. Lipids are the most abundant class, primarily comprising Fatty Acids, Fatty Acyls, and Isoprene lipids. Lipids are involved in signal transduction and energy storage, and are key components of cell membranes, cuticular waxes, and subcutaneous fat [27,28,29]. They also serve as a major carbon source for soil microorganisms [30]. Fatty acids, a type of organic acid, exhibit antimicrobial, antifungal, and antimalarial activities, posing minimal environmental hazards [31]. In summary, PGPR strains induce alterations in the lipid metabolism of alfalfa, which not only support its normal growth and development but also play a crucial role in energy supply, maintenance of cellular structure, and response to environmental stress. In addition to Lipids, Ketones, Aldehydes, and Esters were also identified as significant metabolites in this study. Cheng et al. [31] found that flavonoids were the most abundant class in colored wheat and colored highland barley at different developmental stages, followed by amino acid derivatives, lipids, and nucleotide derivatives, indicating that flavonoids may be a significant contributor to the nutritional components of colored grains. Comparative analysis revealed significant variations in the primary metabolite profiles across different plant species [31]. These disparities likely stem from a combination of genetic traits, environmental conditions, and interactions with the microbial communities. Significant differences in the major metabolite classes can result from variations in plant species and treatment methods.

### 4.2. Differential Metabolites in Response to Different Bacterial Treatments

Following inoculation of alfalfa with single or composite strains, growth promotion occurs via multiple metabolic pathways. Upon strain induction, root-specific metabolites accumulate within the amino acid metabolism and the metabolism of other amino acid pathways, indicating that amino acid metabolic regulation is associated with PGPR-mediated growth promotion. This directly demonstrated the critical role of amino acid metabolic regulation in the PGPR growth promotion mechanism. Amino acid metabolism serves as a critical indicator of plant response to strain induction, thereby influencing plant growth [32]. Glycine and alanine, among others, can promote growth, potentially due to their roles as precursors in biomolecule synthesis or their involvement in energy metabolism [33]. Tyrosine promotes root growth, which in turn affects water uptake. In the study of drought stress in alfalfa, the accumulation of tricarboxylic acid (TCA) cycle intermediates, such as succinate, is closely related to energy optimization and osmotic regulation [34]. In this study, the sustained enrichment of the TCA cycle may reflect that the strain treatment promoted the efficiency of root carbon metabolism, providing an energy basis for nitrogen assimilation and secondary metabolism, indirectly promoting plant growth [35].

The D-amino acid metabolic pathway was enriched in all three treatments. D-amino acids, as bacterial cell wall components and signaling molecules, are involved in the regulation of plant–microbe interactions [36]. Studies have shown that rhizosphere microorganisms (such as rhizobia and dark-septate endophytes) can affect plant immunity and nutrient absorption by regulating D-amino acid metabolism [37]. The enrichment of this pathway in this study may imply that the strain treatment altered the root microecology, promoting the signal transduction of microbial derivatives, which is consistent with the law of microbial community succession and metabolite accumulation in continuous alfalfa cultivation [38]. In this study, the arginine biosynthesis pathway was significantly enriched, and key metabolites L-citrulline and L-arginine were both upregulated. Arginine, as a precursor of polyamines and nitric oxide (NO), plays a core role in plant stress resistance and root development. Studies on rhizobium inoculation of alfalfa have shown that the upregulation of the arginine pathway can enhance antioxidant enzyme activity and ion homeostasis, improving salt stress tolerance [39]. The continuous activation of the arginine pathway in this study may be related to the strain promoting nitrogen metabolism and stress response, which may help improve the antioxidant capacity of alfalfa, reduce the damage of oxidative stress to cells, and maintain normal cell physiological functions, thereby promoting plant growth. Nitrogen is one of the essential macronutrients for plant growth. Improving nitrogen use efficiency provides plants with sufficient nitrogen sources, thereby promoting physiological processes such as protein synthesis and directly stimulating plant development. The citrulline–NO cycle indicates that the plant urea cycle may occur within the roots.

In addition, the induction of PGPR strains leads to alfalfa converting stored organic nitrogen into inorganic nitrogen, thereby improving nitrogen use efficiency. This process may further regulate physiological functions within the plant, synergistically with the urea cycle, promoting alfalfa growth. The lysine degradation pathway was enriched in the MN and MP–N treatments, and this pathway is related to adversity response and reactive oxygen species (ROS) scavenging. In the study of drought stress in alfalfa, lysine degradation products are involved in antioxidant defense and osmotic protection [34]. Similarly, when rhizobia inoculate alfalfa under heavy metal stress, enhanced lysine metabolism can reduce oxidative damage [40]. The enrichment of this pathway in this study may indicate that MN and MP–N treatments more directly activated stress response mechanisms, in contrast to the metabolic preference of MP treatment.

### 4.3. The Primary Metabolic Pathway Through Which PGPR Strains Induce Alfalfa Growth Is Isoflavonoid Biosynthesis

The isoflavonoid biosynthesis pathway was identified as the primary metabolic pathway through which PGPR strains induce alfalfa growth promotion, indicating a key role in plant–microbe interactions. Research indicates that there are more than 30 isoflavonoids in alfalfa, with the most common ones including quercetin, apigenin, and luteolin [41]. In this study, the common differential metabolites across the groups are isoflavonoids, mainly Formononetin, isoformononetin, biochanin A, apigenin, 6″-O-malonylglycitin, and medicarpin 3-O-glucoside-6′-malonate. Analysis of the differential metabolite pathway enrichment maps revealed both unique and shared differential metabolites across the comparison groups, with isoflavonoids consistently identified as key metabolites. This suggests that changes in isoflavonoid levels are not limited to specific treatment groups but significantly impact alfalfa growth under various conditions, highlighting their broad importance in plant–microbe interactions.

Wang Jing et al. [42] detected nine isoflavonoids in alfalfa roots, including apigenin, chickpea sprouts A, daidzein, kaempferol, naringenin, genistein, luteolin, vestitol, and quercetin. The differential metabolites common to all groups in this study were isoflavonoids, with specific compounds identified. Similar to the findings of Wang [43] who detected various isoflavones in alfalfa roots, our results confirm the presence and significance of isoflavonoids in alfalfa, despite some variations in specific types. These differences may be attributed to the specific induction by PGPR, variations in metabolite distribution across different tissues (roots vs. whole plant), or differences in experimental materials, treatments, or detection methods. However, these findings support the existing research on isoflavonoids in alfalfa, further emphasizing their prevalence and importance in this species. Isoflavonoids in plants act as signaling molecules and phytoalexins. As signaling molecules, they induce nodulation in leguminous plants by attracting rhizobia and regulating nitrogen fixation gene expression, thereby enhancing nitrogen utilization [44,45,46].

Furthermore, the metabolic regulatory mechanisms induced by PGPR strains in alfalfa are closely associated with the dynamic changes of metabolites during plant developmental stages. Research indicates significant differences in metabolite accumulation in alfalfa across various developmental stages, including germination, budding, and flowering. Specifically, the leaves during the germination stage exhibit higher levels of flavonoids and amino acids, whereas the accumulation of these metabolites generally decreases during the flowering stage [47,48]. The introduction of PGPR strains modulates amino acid metabolism, such as the upregulation of L-citrulline and L-arginine, and enhances the activity of the tricarboxylic acid cycle. This not only promotes root growth and improves nitrogen utilization but also significantly increases the accumulation of isoflavonoids, including formononetin, isoformononetin, and medicarpin. These isoflavonoid metabolites typically accumulate during plant development, with increased levels as flowering progresses, particularly reaching high levels during the bud and early flowering stages [49]. The induction by PGPR may simulate or enhance the metabolic characteristics of plants at specific developmental stages, such as stress response or reproductive growth. By activating the phenylpropanoid biosynthesis pathway and ABC transporters, it coordinates the synthesis and transport of secondary metabolites, thereby maintaining growth advantages under drought or salt stress conditions [50]. Furthermore, the accumulation of isoflavonoids plays a key role in flower development and stress response. PGPR, by regulating these metabolic pathways, may indirectly influence the transition of alfalfa developmental stages and the synergistic enhancement of stress resistance [51].

These findings suggest that PGPR strains promote alfalfa growth by modulating the isoflavonoid metabolic pathway. The rational use of PGPR strains can increase isoflavonoid content, enhancing stress resistance and nutritional quality. Further research should optimize PGPR strain application to improve alfalfa growth and quality.

## 5. Conclusions

**Figure 9:**
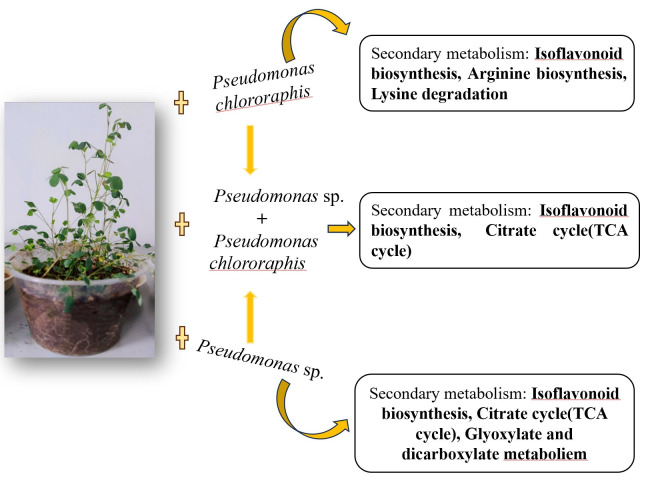
Result schematic diagram.

Utilizing a QTRAP (6500+) platform-based, broad-spectrum targeted metabolomics approach, this study identified, analyzed, and compared the metabolites associated with the growth promotion of alfalfa induced by distinct PGPR strains (*Pseudomonas* sp. and *Pseudomonas chlororaphis* strains). The findings revealed that lipids constitute a major component during the growth of alfalfa. Furthermore, the overlapping differential metabolites within the isoflavonoid biosynthesis pathway, observed during both PGPR-induced and non-induced alfalfa growth, were identified as key metabolites influencing the study’s outcomes. Specifically, the levels of Formononetin, Isoformononetin, Biochanin A, Apigenin, 6″-O-Malonylglycitin, and Medicarpin 3-O-glucoside-6′-malonate were significantly upregulated (Figure 9). This study represents a starting point for future research aimed at investigating metabolites and their mechanisms of action at more advanced stages of plant development (e.g., flowering and fruiting).

## Data Availability

All data generated or analysed during this study are included in this published article and its (Supplementary Information files).

## Supplementary Materials

The following supporting information can be downloaded at: https://www.mdpi.com/article/10.3390/metabo15100652/s1. Table S1: Inter-group correlation; File S1: Growth-Promoting Attributes and Methodology.
