# Supplementary material for: Metabolic Characteristics of PGPR-Induced Growth Promotion in Alfalfa (Medicago sativa L.)
Source: Metabolites. 2025 Sep 30;15(10):652. doi: 10.3390/metabo15100652 (PMC12566109; doi:10.3390/metabo15100652)
Supplement: Supplementary file 1 [file metabolites-15-00652-s001.zip › Word S1 Growth-Promoting Attributes and Methodology.pdf]

1 Culture medium used in the experiment

The culture media employed in the experiments included a nitrogen-free medium (NFM) for the isolation of nitrogen-fixing bacteria; Mongina medium, which contains egg yolk lecithin as the sole phosphorus source, for the isolation of organic phosphorus-solubilizing bacteria; and the National Botanical Research Institute's Phosphate (NBRIP) medium, with $Ca_3(PO_4)_2$ as the sole phosphorus source, for the isolation of inorganic phosphorus-solubilizing bacteria. Additionally, Luria-Bertani (LB) broth was used for the observation and preservation of bacterial colony morphology.

2 Isolation and Screening of PGPR Strains

Soil adhering to the rhizosphere roots was gently dislodged and surface soil was collected using a brush. A 10 g sample of soil was weighed and transferred into a 150 mL conical flask, to which 90 mL of sterile physiological saline (0.85%) was added. The mixture was shaken on a rotary shaker for 30 minutes to obtain a 10^{-1} dilution. Serial dilutions were prepared to generate 10^{-2}, 10^{-3}, 10^{-4}, and 10^{-5} dilutions. Aliquots of the 10^{-3}, 10^{-4}, and 10^{-5} dilutions were plated onto NFM, NBRIP, and Mung Bean Agar media using the spread plate method, with three replicates per concentration. The inoculated plates were inverted and incubated at 28°C for 3 days to observe colony development. Distinct colonies exhibiting rapid growth and diverse morphologies were selected and streaked onto fresh media for purification, repeated multiple times until pure cultures free of contaminants were obtained. The purified strains were inoculated onto LB agar slants and stored at 4°C for future use.

3 Analysis of the Plant Growth-Promoting Traits of PGPRs

(1) Screening of Nitrogen-Fixing Strains

Bacterial isolates were inoculated onto NFM medium and incubated at 28°C, with periodic observation of colony development. The presence of colonies was indicated by a "+", reflecting nitrogen fixation capability. Starting from the second day of streaking, colonies appearing were recorded as "+++++". Each day of delay in colony emergence resulted in the removal of one "+". Strains that failed to produce colonies were marked as "–". This method facilitated the selection of strains with faster growth rates.

Table S1 Nitrogen fixing capacity of PGPR strains

strains	Nitrogen fixation capability	strains	Nitrogen fixation capability
N-ASC	++++	OP-SSBL	++++

(2) Phosphate Solubilization Ability

The phosphate solubilization zone diameter (D) and colony diameter (d) were measured using the halo method. Quantitative determination of soluble phosphorus was performed via molybdenum blue colorimetric assay.

Table S2 Ability of PGPR to dissolve organic phosphorus

strains	Organic phosphorus solubility（μg/mL）	D/d
OP-SSBL	20.08±2.18	1.13±0.05d
N-ASC	20.12±1.99	1.93±0.25

Table S3 Ability of PGPR to dissolve inorganic phosphorus

strains	Inorganic phosphorus solubilization quantity（μg/mL）	D/d
N-ASC	79.26±0.77	1.26±0.22
OP-SSBL	76.09±4.04	1.34±0.10

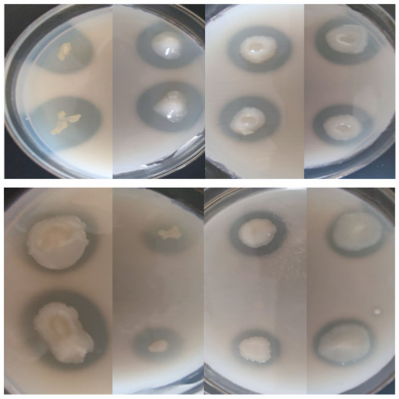

Figure S1 Phosphate solubility of strains

(3) Indole-3-Acetic Acid (IAA) Secretion

The ability of strains to produce IAA was assessed using both Salkowski's colorimetric method for quantitative analysis and a qualitative color development assay, following established protocols.

Table S4 Ability of PGPR to secrete IAA

strains	IAA concentration (μg/ml)	Chromogenic reaction	strains	IAA concentration (μg/ml)	Chromogenic reaction
N-ASC	24.31±0.05	+++	OP-SSBL	24.83±0.03	+++

4 Molecular Identification of the Excellent Strain

Total genomic DNA was extracted from bacterial cultures using the Ezup bacterial genomic DNA extraction kit (purchased from Shanghai Shenggong Biological Engineering Technology Co., Ltd.) with bacterial suspension as the template. Universal bacterial primers 27F (5'-AGTTTGATCMTGGCTCAG-3') and 1492R (5'-GGTTACCTTGTTACGACTT-3') were employed for PCR amplification of the 16S rDNA gene, synthesized by Shanghai Shenggong Biological Engineering Technology Co., Ltd. The PCR reaction mixture consisted of 25 μL Taq

PCR premix, 1 μL DNA template, 2 μL primer F, 2 μL primer R, and 20 μL sterile deionized water. The amplification conditions were as follows: initial denaturation at 95°C for 3 minutes; denaturation at 95°C for 30 seconds; annealing at 55°C for 30 seconds; extension at 72°C for 1 minute; and a final extension at 72°C for 10 minutes. The obtained sequences were subjected to similarity searches using MegaBLAST in the NCBI database (http://www.ncbi.nlm.nih.gov/BLAST/), and homology comparisons were performed with reported bacterial 16S rDNA sequences. Phylogenetic analysis was conducted by constructing a distance tree using MEGA 7.0 software, employing the Neighbor-Joining method for systematic evolutionary analysis.

Table S5 Identification of superior PGPR strains

ID	length	homologous strains	Similarity index
OP-SSBL	1412	*Pseudom*onas chlororaphis	96.48%
N-ASC	1362	*Pseudomonas* sp.	96.77%

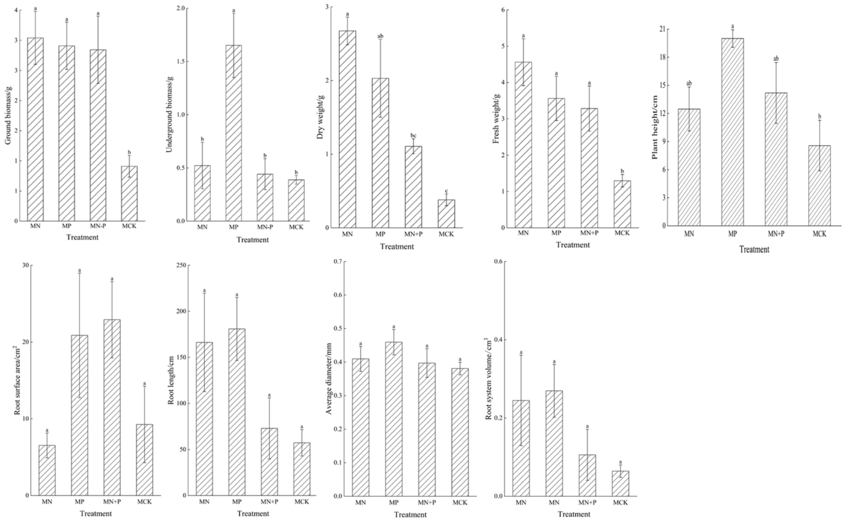

Figure S2 The biological characteristics of *Medicago sativa*. Different letters indicate significant differences (P<0.05).
